# A Dissolved Oxygen Threshold for Shifts in Bacterial Community Structure in a Seasonally Hypoxic Estuary

**DOI:** 10.1371/journal.pone.0135731

**Published:** 2015-08-13

**Authors:** Rachel L. Spietz, Cheryl M. Williams, Gabrielle Rocap, M. Claire Horner-Devine

**Affiliations:** 1 School of Aquatic and Fishery Sciences, University of Washington, Seattle, Washington, United States of America; 2 School of Oceanography, University of Washington, Seattle, Washington, United States of America; Auckland University of Technology, NEW ZEALAND

## Abstract

Pelagic ecosystems can become depleted of dissolved oxygen as a result of both natural processes and anthropogenic effects. As dissolved oxygen concentration decreases, energy shifts from macrofauna to microorganisms, which persist in these hypoxic zones. Oxygen-limited regions are rapidly expanding globally; however, patterns of microbial communities associated with dissolved oxygen gradients are not yet well understood. To assess the effects of decreasing dissolved oxygen on bacteria, we examined shifts in bacterial community structure over space and time in Hood Canal, Washington, USA−a glacial fjord-like water body that experiences seasonal low dissolved oxygen levels known to be detrimental to fish and other marine organisms. We found a strong negative association between bacterial richness and dissolved oxygen. Bacterial community composition across all samples was also strongly associated with the dissolved oxygen gradient, and significant changes in bacterial community composition occurred at a dissolved oxygen concentration between 5.18 and 7.12 mg O_2_ L^-1^. This threshold value of dissolved oxygen is higher than classic definitions of hypoxia (<2.0 mg O_2_ L^-1^), suggesting that changes in bacterial communities may precede the detrimental effects on ecologically and economically important macrofauna. Furthermore, bacterial taxa responsible for driving whole community changes across the oxygen gradient are commonly detected in other oxygen-stressed ecosystems, suggesting that the patterns we uncovered in Hood Canal may be relevant in other low oxygen ecosystems.

## Introduction

Globally, over a quarter-million square kilometers of marine ecosystems are threatened by low dissolved oxygen (DO) levels, or hypoxia, which can result in the exclusion or death of resident macroorganisms creating so-called ‘dead-zones’ [1]. The number of hypoxic coastal areas has grown at an exponential rate of 5.54% year^-1^ [2], largely due to increased eutrophication [3,4]. Seasonal hypoxia is detrimental to coastal ecosystems and is known to have negative effects on fish [5], crustacea [6], benthic invertebrate communities [7], and trophic interactions and energy flow [8]. However, Bacteria and Archaea remain active in hypoxic waters and perform important functions in mineralization of organic matter and other biogeochemical cycling [1,9].

Extensive research has gone into understanding the thresholds of hypoxia for management strategies aimed at avoiding catastrophic collapses of ecosystems. In a review of over 800 published experiments, Vaquer-Sunyer and Duarte [2] report that published thresholds of hypoxia span a broad range from 0.29 mg O_2_ L^-1^ to 4 mg O_2_ L^-1^, but on average, sublethal effects on marine benthic macroorganisms occur when DO concentration drops below 2.61 ± 0.17 mg O_2_ L^-1^ and lethal effects occur at 2.05 ± 0.09 mg O_2_ L^-1^, with many taxa of Crustacea and Fishes demonstrating negative effects of hypoxia near 4 mg O_2_ L^-1^. It is worth noting, however, that thresholds for hypoxia likely differ between coastal open ocean hypoxic zones, as the organisms in each may be adapted to different durations of hypoxia [10]. Lacking from our current knowledge is whether a threshold of hypoxia for bacterial communities exists, and, if so, at what DO concentration do bacterial communities experience taxonomic and functional shifts.

Understanding the effect of hypoxia on lower trophic levels including plankton assemblages is crucial for elucidating whole ecosystem effects and interactions among trophic levels. Many microbial studies have focused on truly oxygen deficient zones where anaerobic metabolisms dominate including the Eastern Tropical South Pacific [11] and North Pacific [12], Arabian Sea [13,14], Cariaco Basin [15,16], Baltic Sea [17], Black Sea [18–20], and Saanich Inlet [21]. A recent meta-analysis examining global patterns of microbial community composition in oxygen-minimum zones found that particular bacterial and archaeal taxa, specifically Proteobacteria, Bacteroidetes, marine group A, Actinobacteria, and Planctomycetes, are common in suboxic waters and that these taxa tend to co-occur in a non-random pattern along the oxycline [22]. Only some of the members of these suboxic-associated phyla are known to use electron acceptors alternative to oxygen, which might suggest that mechanisms other than oxygen-depletion drive changes in the bacterial community. Though these previous studies have demonstrated that particular bacterial taxa persist in oxygen-minimum zones, the threshold concentration of DO at which bacterial communities change remains unclear. The bacterial community that exists at dysoxic (0.66–2.96 mg O_2_ L^-1^) and suboxic (0.03–0.66 mg O_2_ L^-1^) levels has the potential to become increasingly important in global biogeochemical cycles as oxygen-minimum zones in both the open and coastal ocean continue to expand with climate change [23], thus emphasizing the importance of defining a threshold DO concentration for Bacteria.

**Fig 1:**
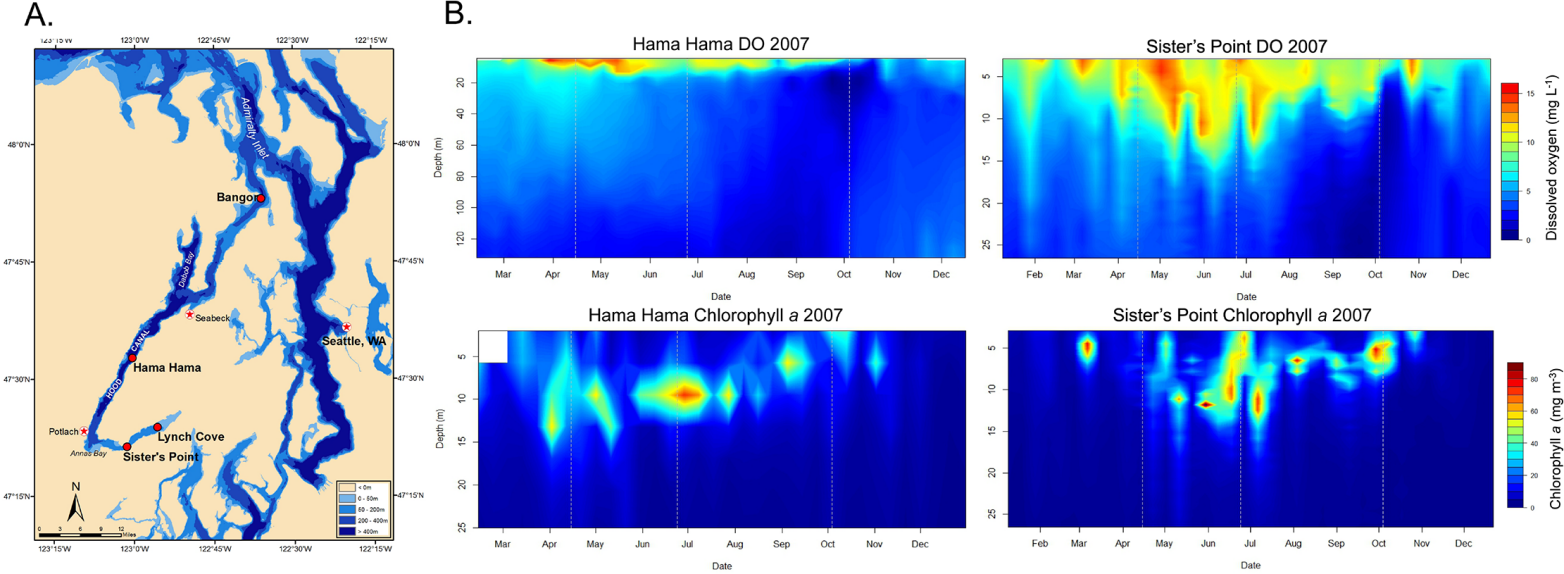
Geography and biogeochemical conditions in Hood Canal, WA, USA in 2007. (A) Map of sampling stations within Hood Canal, WA, USA. (B) Contour plots showing range of dissolved oxygen (DO) and chlorophyll *a* concentrations at Hama Hama and Sister’s Point stations in Hood Canal, WA. Data for high-resolution depth profiles were collected by Oceanic Remote Chemical Analyzer buoys maintained by the Northwest Association of Networked Ocean Observing Systems (http://www.nanoos.org). Note changes in scale on both the x- and y-axes.

We examined spatial and temporal variation in bacterial communities in Hood Canal, WA, USA between April and October of 2007. Hood Canal is a long and narrow glacial fjord located 80 miles west of Seattle, Washington, USA (Fig 1A), that seasonally experiences periods of low and even completely depleted DO concentrations as a result of naturally occurring physical and hydrographical conditions [24]. This system has relatively small-scale, enclosed circulation patterns and rapidly changing abiotic conditions. Throughout the year, Hood Canal remains highly stratified, which limits the movement of oxygenated waters [25]. Furthermore, human activity over the past few decades has exacerbated nutrient loading particularly in the southern reaches of Hood Canal [26], in turn increasing the frequency and duration of recent hypoxia events [27]. Fish and macroinvertebrates in Hood Canal are directly affected by hypoxia through mortality or distributional shifts [28,29]. Particularly significant fish kill events near the southern reaches of the canal have occurred in the Falls of 2002, 2003, 2004, and 2006 as well as in the Spring of 2006 when the oxygen-deprived deep-water mass shoaled to the surface as a result of a combination of southerly winds and fresh-water intrusion from the Puget Sound at the North end of the canal [30]. Additionally, mesozooplankton assemblages in Hood Canal demonstrated altered composition and behavior when DO dropped below ~1.5 mg O_2_ L^-1^ [31]. These shifts have implications for predator-prey dynamics and trophic energy transfer and suggest that hypoxia may affect the entire biologic community in Hood Canal. We examined the bacterial communities associated with the seasonal variability in abiotic factors in Hood Canal, including depleted DO concentration. We hypothesized that bacterial communities in Hood Canal demonstrate non-random changes in composition and taxa richness related to variation in abiotic factors, and that these community patterns are associated particularly with changing levels of DO.

## Methods

### Sample collection

High resolution depth profiles of DO and chlorophyll *a* for two mid Hood Canal, WA stations, Hama Hama and Sister’s Point, were obtained via Oceanic Remote Chemical Analyzer (ORCA) buoys maintained by the Northwest Association of Networked Ocean Observing Systems (http://www.nanoos.org) [32]. Water samples were collected during April, June, and October of 2007 into Niskin bottles mounted on a rosette, equipped with a CTD (conductivity, temperature, and depth) sampler (Sea-Bird Electronics, Bellevue, WA, USA) that measured in-situ temperature and salinity. April and October samples were collected from two stations near the middle of Hood Canal: Hama Hama and Sister’s Point. During the June sample collection, two additional stations (Bangor and Lynch Cove) were added in order to survey a full north to south transect of the Canal. At each sampling station and time, samples were collected both at five meters depth (herein referred to as surface samples) as well as ten meters above the bottom substrate (herein referred to as deep samples). These so-called deep samples varied in depth from 140−145 m at Hama Hama, 125 m at Bangor, 40−50 m at Sister’s Point, and 12 m at Lynch Cove, as the depth of the water column decreases near the southern reaches of the canal. The timing of the collection points were intended to coincide with the late spring algal bloom (April), subsequent die-off (June), and a smaller autumn algal bloom (October), which affect DO concentration and nutrient availability (S1 Table). A field permit was not required for the sampling in this study, as there are no protections in place for the waters or organisms that would be impacted by our sampling and sampling did not affect any endangered or protected species.

Immediately upon retrieval of the Niskin bottles, DO samples were collected using surgical tubing into calibrated glass bottles with ground glass stoppers and were analyzed using a modified Winkler titration [33]. The remaining water was used for additional measurements of biotic and abiotic parameters. For inorganic nutrients, 50 mL of water was passed through a 0.45-μm syringe filter and immediately frozen until being analyzed at the University of Washington’s Oceanography Marine Chemistry Lab for NO_3_^-^ (detection limit 0.15 μM), NO_2_^-^ (detection limit 0.02 μM), NH_4_^+^ (detection limit 0.12 μM), PO_4_^3-^ (detection limit 0.03 μM), and SiOH_4_ (detection limit 0.59 μM) following the Protocols for the Joint Global Ocean Flux Survey (JGOFS) Core Measurements (1994) [34]. To measure bacterial abundance, 5 mL of water was preserved in formaldehyde with a final concentration of 1% and stored at -80°C until 0.5–1.0 mL aliquots were filtered onto 0.22-μm polycarbonate filters and stained with 4’,6-diamidino-2-phenylindole (DAPI). The DAPI filters were viewed under a Nikon Eclipse 80i with UV light from an X-cite Series EXFO to count the number of cells present in fifteen randomly selected fields using NIS-Elements BR 3.0 software then averaged to quantify the total bacterial abundance. Principal components analyses and all other statistical analyses were conducted in *R* using the *vegan* package (http://cran.r-project.org/web/packages/vegan/index.html), unless noted otherwise. Metadata are provided in S1 Table.

### DNA extraction and processing

For bacterial community analyses, 300 mL of water was filtered onto 0.22-μm Supor filters and preserved with 3 mL of solution of 0.5 M NaCl, 10 mM Tris, and 100 mM EDTA, then immediately frozen at -80°C. A Qiagen DNeasy Tissure Kit was used to extract DNA from the filters following the manufacturer’s instructions for extraction from gram-negative Bacteria (Qiagen Valencia, CA). The DNA extracts were sent to MBL’s Keck Facility for PCR amplification and sequencing on the GS-FLX Titanium 454 platform (see Sogin, et al., 2006 for previously described methods [35]). Briefly, the V6 region of the 16S rRNA gene was amplified via polymerase chain reaction (PCR) using a pool of bacterial specific primers, excluding amplification of Archaea (see Huse, et al., 2010 for description of V6 primer pool [36]). Sequencing was performed in conjunction with the International Census of Marine Microbes (ICoMM) project (http://icomm.mbl.edu), which uses a standardized 454 pyrosequencing pipeline to sequence marine microbial samples from across the globe [37]. All sequences were deposited in GenBank Sequence Read Archives (www.ncbi.nlm.nih.gov) under accession number SRP001226.

As part of the ICoMM project, the sequence data were initially analyzed using a standardized pipeline [35,36,38]. Sequences were pre-clustered using a single-linkage algorithm to smooth sequencing errors and reduce noise. Sequences were then clustered using average-linkage into operational taxonomic units (OTUs) based on 97% sequence identity. Prior to community analyses, we removed sequences that were highly similar to chloroplast sequences.

### Statistical analyses

Abiotic factors, including NO_3_^-^, NO_2_^-^, NH_4_^+^, PO_4_^3-^, SiO_4_^2-^, salinity, temperature, and DO concentration, were log-transformed prior to analyses to meet assumptions of normality and homogenization of variance. Euclidean distance between samples was calculated for environmental variables [39]. Principal components analysis (PCA) was performed to assess dominant trends in environmental variables between samples across depths, sites, and seasons. A Monte Carlo randomization test was used to test the significance of each principal component at an alpha level of 0.05.

To minimize the effect of sequencing effort on richness estimates, all samples were randomly subsampled down to 3663 sequences, which was the number of sequences of the sample with the fewest number of sequences (Table 1), the richness measures were calculated, then the process was repeated and averaged over 1000 iterations. Observed taxa richness, or the number of OTUs identified at the pre-defined sequence identity, as well as the Chao richness estimator were calculated using the subsampling method and averaged over 1000 iterations (Table 1). We used multiple regression modeling with a requirement of α < 0.05 for a variable to enter the model to determine which abiotic factors best described the changes in taxa richness, evenness (Pielou’s J), and proportion of functional groups across all samples.

**Table 1:** Number of sequences obtained in each sample, as well as the observed and Chao estimates of taxa richness and Pielou’s J index of taxa evenness for all Hood Canal samples based on an operational taxonomic unit cutoff of 97% sequence identity.

Sample	Location	Season	Depth	Number of sequences	Observed richness	Chao	Pielou's J
HH_APR_S	Hama Hama	April	Surface	14743	155	376	0.58
HH_APR_D	Hama Hama	April	Deep	14897	512	980	0.75
SP_APR_S	Sister’s Point	April	Surface	10482	173	353	0.68
SP_APR_D	Sister’s Point	April	Deep	14739	554	1303	0.75
BA_JUN_S	Bangor	June	Surface	14019	239	430	0.71
BA_JUN_D	Bangor	June	Deep	11108	302	595	0.72
HH_JUN_S	Hama Hama	June	Surface	6729	262	537	0.73
HH_JUN_D	Hama Hama	June	Deep	11540	467	840	0.74
SP_JUN_S	Sister’s Point	June	Surface	7077	254	516	0.70
SP_JUN_D	Sister’s Point	June	Deep	10368	546	1219	0.74
LC_JUN_S	Lynch Cove	June	Surface	11595	193	342	0.72
LC_JUN_D	Lynch Cove	June	Deep	8379	339	600	0.70
HH_OCT_S	Hama Hama	October	Surface	3663	447	1043	0.71
HH_OCT_D	Hama Hama	October	Deep	11445	498	966	0.75
SP_OCT_S	Sister’s Point	October	Surface	4446	238	576	0.62
SP_OCT_D	Sister’s Point	October	Deep	9795	590	1193	0.76

BA = Bangor; SP = Sister’s Point; HH = Hama Hama; LC = Lynch Cove; APR = April; JUN = June; OCT = October; S = Surface; D = Deep.

We used multivariate statistical analyses to identify important drivers of the relationship between bacterial community composition and environmental factors. We selected a Sorensen abundance-based dissimilarity measure because it incorporates probability of detection of taxa shared between two samples to account for under-sampling, which is common when studying microbial communities [40]. Similarly to the alpha diversity calculations, the bacterial community matrix was randomly subsampled to the number of sequences of the smallest sample then the Sorensen abundance-based similarity was calculated between samples to account for sequencing effort bias [41,42] and averaged over 1000 iterations.

Analysis of similarity (ANOSIM) tests examined the effects of factors including depth, site, season, or DO (high or low) on bacterial community composition of each sample and significance was tested using 999 random permutations of the community composition dataset. A non-parametric iterative *BIO-ENV* analysis was employed to select the set of abiotic variables most correlated with changes in bacterial community composition [43].

Threshold Indicator Taxa Analysis (TITAN) detects changes in taxa distributions along an environmental gradient that exists over space and/or time [44]. The analysis assesses the synchrony among taxa change points as evidence for community thresholds. Prior to TITAN, the sixteen Hood Canal samples were randomly subsampled to the smallest number of sequences present in a single sample (3663 sequences). In order to accurately calculate an indicator value (IndVal) for each taxon, the taxon must be present in three or more samples. Therefore, taxa with narrow distributions (present in less than three samples) were eliminated from each subsampled dataset. Finally, all bacterial abundance data were log-transformed to down-weight highly abundant types. TITAN was run in *R* using a minimum split of three samples, 100 random permutations to calculate IndVal p-values, and 100 random permutations for the bootstrapping calculation. Subsampling and TITAN analysis was repeated ten times to confirm that stochastic changes in sampling do not affect the results of TITAN.

To validate the DO threshold identified by TITAN, we used a non-parametric ANOSIM test grouping samples either by depth or DO level split at either 4 mg O_2_ L^-1^ or 6 mg O_2_ L^-1^. The R-values from ANOSIM, which describes how well samples group by factor based on community composition, were compared to determine the best factor for grouping.

Finally, hierarchical clustering using average linkage was used to cluster samples based on dissimilarity of community composition quantified by the Sorensen abundance-based estimator to identify the DO concentration at which the community composition changed significantly. All three analyses, TITAN, ANOSIM, and clustering were performed in *R* using scripts from Baker and King [44]; *vegan*, *cluster*, and *pvclust* packages and scripts written for this study, which are available upon request.

A similarity percentage (SIMPER) analysis was used to identify bacterial taxa contributing most to the similarity within groups of samples defined by DO and thus identify taxa strongly associated with either high or low DO [45]. SIMPER analysis identifies taxa that contribute the most to the similarity in community composition between samples of the same group, or similarly, taxa that contribute the most to dissimilarity in community composition across groups of samples. Thus, the taxa identified by SIMPER are significant indicators of a particular factor, in this case low DO. Dissolved oxygen is usually higher in surface waters than in deep waters, so to separate a depth effect on taxa distribution from an oxygen effect, the SIMPER analysis was run a second time using sampling depth as a factor rather than oxygen level. To test the significance of an oxygen effect over a depth effect, a generalized linear model was run using an intercept of 0 and a slope of 1, which would imply a null hypothesis that there is no difference between a depth or DO effect. Studentized residuals were calculated for each taxon, and a Bonferroni correction was applied to calculate a 95% confidence interval because of the high number of taxa or data points. Taxa that demonstrated a significant effect of DO level rather than sampling depth were those that fell above the 95% confidence interval, and thus had a studentized residual greater than 4.2169. For taxa identified to be significant indicators of high or low DO, their taxonomy was further resolved by using the V6-16S rRNA gene sequences to perform both a blastn search against the NCBI nr/nt database in (http://blast.ncbi.nlm.nih.gov/Blast) and also by aligning the V6-16S rRNA gene sequences against the arb-silva database using the online SINA v1.2.11 aligner (http://www.arb-silva.de/aligner/) [46]. All alignments were performed in June and July of 2014.

## Results

### Environmental context

Using high-resolution measurements of DO and chlorophyll *a* (Fig 1B), it was apparent that patterns of hypoxia in 2007 were not as extreme as in past years when low DO had caused fish kills. Yet, our samples captured a wide range of abiotic conditions and likely represent typical seasonal variability in Hood Canal. Water samples were collected from Hama Hama and Sister’s Point (Fig 1A) in April, directly following the major spring phytoplankton bloom as indicated by the heightened level of chlorophyll *a* in the surface water layer at both stations, marked by high DO concentrations (12.32−13.18 mg O_2_ L^-1^) and low nutrient concentrations in the surface waters (0.03−0.73 μM NO_3_^-^) and low DO concentrations (3.59−3.94 mg O_2_ L^-1^) and high nutrient concentrations in deep waters (Fig 1B). June samples were collected at the end of the phytoplankton bloom, and thus the surface waters were characterized by slightly lower DO (8.66−10.25 mg O_2_ L^-1^) and nutrient concentrations than in April. At the same time, the deep waters contained low DO (2.95−7.12 mg O_2_ L^-1^) and high nutrient concentrations. October samples were collected after a lesser fall phytoplankton bloom. Dissolved oxygen and nutrient concentrations in October were in the mid to high range, relative to previous months, in both water depths, which were less stratified than earlier in the year (S2 Fig and S1 Table). Bacterial abundances across all stations and time points ranged from 2.16 × 10^5^ cells mL^-1^ to 2.70 × 10^6^ cells mL^-1^, with an average abundance of 8.46 × 10^5^ cells mL^-1^ across all samples.

**Fig 2:**
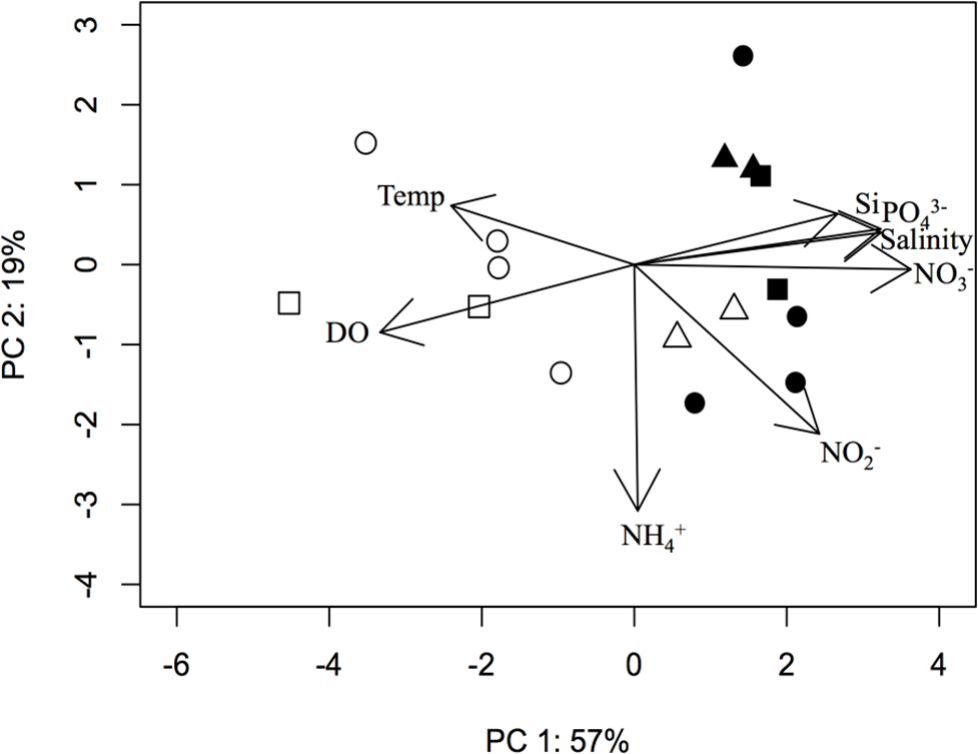
Principal components analysis of environmental characteristics of the sixteen samples ordinated based on Euclidean distance calculated from environmental factors. Points are coded by water depth as either deep (filled symbols) or surface (open symbols) and by season as April (square), June (circle), or October (triangle). Nitrate, phosphate, salinity, and dissolved oxygen best explain the separation of samples along axis one, and ammonium alone explains variation along axis two based on the variable loadings on each principal component.

Using the environmental variables measured at the time of water sample collection, a principal components analysis (PCA) was conducted to visualize abiotic factors driving the differences among samples (Fig 2). Nitrate, DO, salinity, and phosphate were most correlated with PCA axis one, which explained 57% of the total measured abiotic variation among samples. Samples appear to fall into two groups along axis one, mostly described by depth. An additional 19% of the total variation in abiotic parameters was explained by PCA axis two, which was most correlated with ammonium concentration.

### Bacterial alpha diversity in Hood Canal, WA

**Fig 3:**
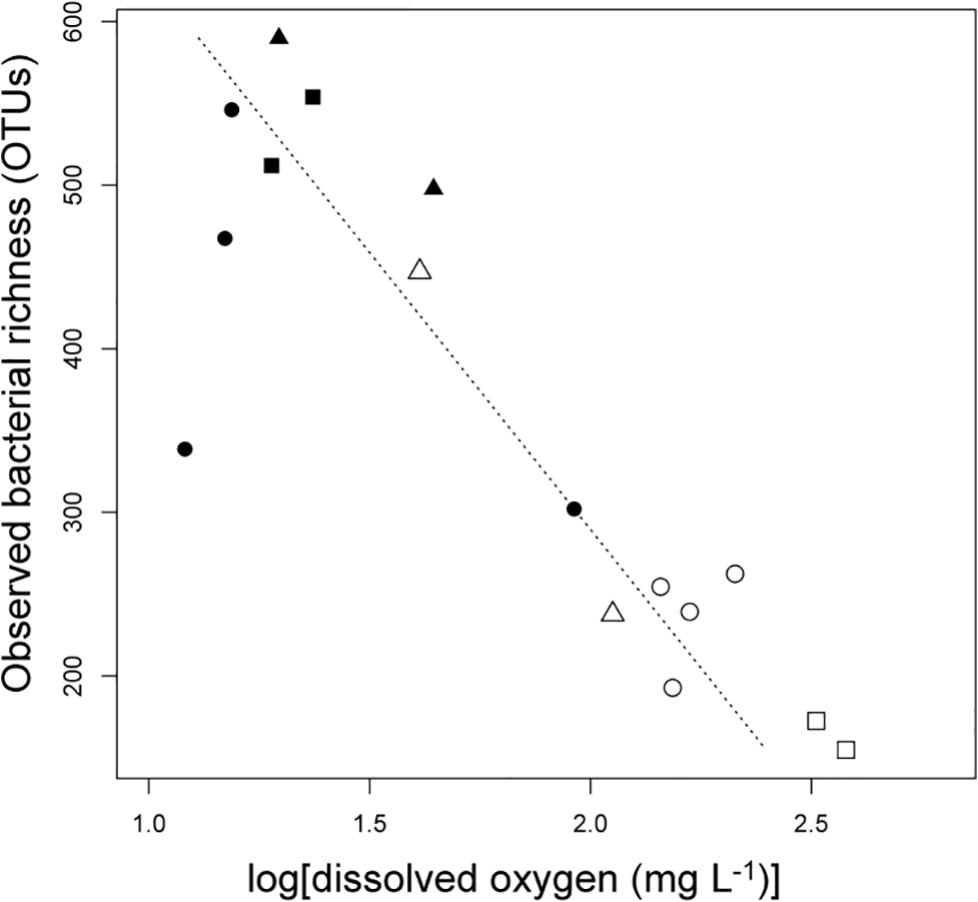
Bacterial alpha-diversity varies by DO. A significant relationship exists between DO concentration and bacterial taxa richness. Points are coded by water depth as either deep (filled symbols) or surface (open symbols) and by season as April (square), June (circle), or October (triangle). Dashed line represents the best fit from the linear regression model of bacterial richness as a function of dissolved oxygen (adjusted R^2^ = 0.7379, p < 0.01).

Observed bacterial taxa richness, or the number of OTUs (defined by 97% sequence similarity clusters) recovered, in Hood Canal ranged from 155 OTUs to 590 OTUs per sample and was significantly higher in the deep-water samples than in the surface water samples by an average factor of two (F_1,14_ = 22.55; p < 0.01) (Table 1, Table 2, Fig 3). Neither station (horizontal space) nor season had a significant effect on bacterial richness (results not shown). By applying a forward-selection multiple regression model using all abiotic variables measured in this study, DO concentration alone best explained the variation in observed bacterial richness across samples (adjusted R^2^ = 0.738, p < 0.01, Fig 3). Taxonomic richness peaked at a DO concentration of 4 mg O_2_ L^-1^ (Fig 3).

**Table 2:** Continuous abiotic variables affect the diversity and composition of bacterial communities in Hood Canal, WA, USA (α = 0.05 for all tests). The strength of richness and evenness, measured by Pielou’s J, models was assessed by the adjusted R^2^ value from multiple linear regressions, and the strength of the composition models was assessed by the Spearman’s rho correlation coefficient from BIO-ENV analyses.

Response	Classification	Model	Adjusted R^2^	Spearman's rho
Richness	Bacterial domain	DO(−)	0.738	
	Alphaproteobacteria	Sal(+)	0.585	
	Flavobacteria	DO(−)	0.705	
	Gammaproteobacteria	DO(−)	0.759	
Pielou's J	Bacterial domain	PO_4_(+)	0.524	
	Alphaproteobacteria	NO_3_(−), Si(−)	0.837	
	Flavobacteria	PO_4_(+), Sal(+), NO_3_(−)	0.854	
	Gammaproteobacteria	DO(−)	0.429	
Composition	Bacterial domain	DO		0.732
	Alphaproteobacteria	DO, PO_4_		0.692
	Flavobacteria	DO		0.765
	Gammaproteobacteria	DO		0.792

Across all samples, over 57% of the sequences clustered into only 42 unique OTUs (defined by 97% sequence identity), while less than 2% of the sequences clustered into over 2300 unique OTUs. The abundant OTUs were typically widespread across all the samples, whereas each of the less abundant OTUs was detected in one or two samples. Uneven distribution of individuals into taxa is not unusual in bacterial communities [35], which we further explored by calculating an index of taxon evenness, Pielou’s J (Table 1) that provided an additional window into bacterial community structure. Evenness of taxa was higher in deep waters than in the surface water (F_1,14_ = 9.517, p < 0.01), while neither season nor sampling site had an effect on the evenness of the bacterial community (Table 2). Using multiple regression modeling, taxa evenness was best explained by PO_4_^3-^ (adjusted R^2^ = 0.524, p < 0.01, Table 2).

**Fig 4:**
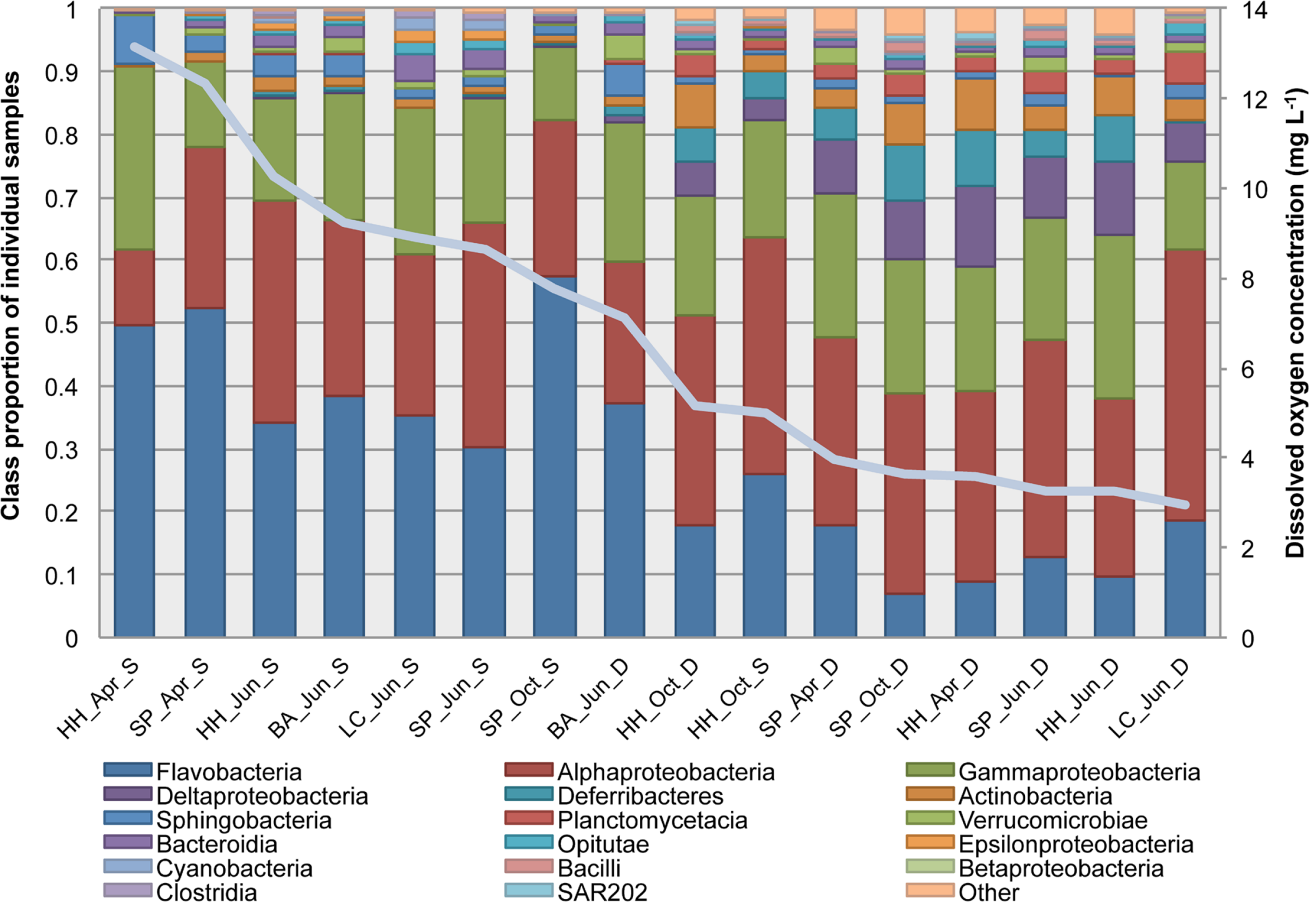
Relative proportions of the most abundant classes change across a dissolved oxygen gradient and are significantly different when grouped by sampling depth. The samples are ordered by dissolved oxygen concentration (solid line). Abbreviations: Hama Hama (HH), Sister’s Point (SP), Bangor (BA), Lynch Cove (LC), April (APR), June (JUN), October (OCT), Surface (S), Deep (D). Classes that comprised less than 1% of the sample’s total community were condensed into the “other” category.

Although an uneven distribution of individuals into taxa is common in bacterial communities [35], changes in the relative proportion of the dominant classes provide insight into factors impacting bacterial community structure (Fig 4). Sampling depth explained a significant amount of the variation in relative proportion of the fifteen most abundant classes across samples (G-test; G = 37.585, p < 0.01), whereas season or horizontal space had no significant effect (alpha < 0.05). Of the most abundant bacterial classes, the majority of sequences recovered from all samples belonged to three classes: *Flavobacteria*, *Alphaproteobacteria*, and *Gammaproteobacteria*. In surface waters these classes comprised 88.5% of sequences, whereas in deep waters they comprised only 68.7% of sequences, which demonstrates higher taxa evenness in deep-water samples. The proportions of *Alphaproteobacteria* and *Gammaproteobacteria* were not significantly different between surface and deep waters; however, the proportion of Flavobacterial sequences was significantly higher in the surface water samples compared to deep-water (F_1,14_ = 21.463; p < 0.01). The majority of *Flavobacteria* detected in the Hood Canal surface samples are members of the Flavobacteriaceae family, which are known to be highly associated with algal particles and are important players in organic matter degradation as part of the microbial loop [47,48]. The high relative abundance of Flavobacteriaceae in the surface water samples of Hood Canal reflect the considerable algal bloom dynamics in the estuary and a recent pulse of organic matter particles into the euphotic zone. The five most abundant taxa in each sample included common, ubiquitous marine bacterial taxa such as those belonging to SAR11, SAR86, Flavobacteriaceae, Thalassobacter, SAR406, SAR324, and Rhodobacteriacaea. However, as DO concentration decreased across samples, the relative abundances of the dominant types changed as well (Fig 4).

### Patterns of functionally relevant taxa

In order to evaluate potential functional changes in the bacterial communities, we examined changes in the relative abundances of several groups of bacteria in our dataset known to be important in specific biogeochemical processes (Table 3). Two groups of ammonia oxidizing bacteria (AOB), *Nitrosomonas* and *Nitrosococcus*, were detected in Hood Canal. The relative abundance of AOB was higher in deep water than surface water (F_1,14_ = 18.98, p < 0.01) and also demonstrated a significant negative relationship with DO concentration (adjusted R^2^ = 0.5765, p < 0.01). Given that we used bacterial-specific 16S rRNA primers, we did not detect ammonia-oxidizing Archaea, which are also known to inhabit Hood Canal [9]. The relative abundance of two groups of nitrite-oxidizing bacteria (NOB) in Hood Canal, *Nitrospira* and *Nitrospina*, was also significantly higher in the deep water than in the surface water (F_1,14_ = 24.9, p < 0.01). Additionally, the relative abundance of NOB was not only strongly associated with a decrease in DO but also with decreased NH_4_^+^ concentration and temperature (adjusted R^2^ = 0.9315, p < 0.01). There were no significant patterns of increased *Cyanobacteria* abundance by water depth, sampling season, or sample site; however, the relative abundance of this photosynthetic phylum was strongly associated with higher temperatures (adjusted R^2^ = 0.8812, p < 0.01). The relative abundance of methylotrophic bacteria in Hood Canal, including Methylobacteriaceae, Methylococcaceae, Methylocystaceae, and Methylophilaceae, also demonstrated no significant patterns across water depths, season, or sampling site but their abundances did increase with decreasing DO and higher temperature (adjusted R^2^ = 0.5984, p < 0.01).

**Table 3:** Abiotic factors affect the average proportion of various bacterial functional groups. A forward selection stepwise multiple linear regression modeling approach was employed with a requirement of p < 0.05 to enter the model.

Functional group	Total no. of reads	Average proportion*	Model	Adjusted R^2^
Nitrite oxidizers	2484	0.0136	DO(−), NH_4_(−), Temp(−)	0.9315
Ammonia oxidizers	300	0.0012	DO(−)	0.5765
Methylotrophs	701	0.0044	DO(−), Temp(+)	0.5984
Cyanobacteria	2241	0.0136	Temp(+)	0.8812

(+) indicates a significant positive association (p < 0.01)

(−) indicates a significant negative association (p < 0.01)

*Average proportion of the functional group within each sample.

### A bacterial community threshold for DO

**Fig 5:**
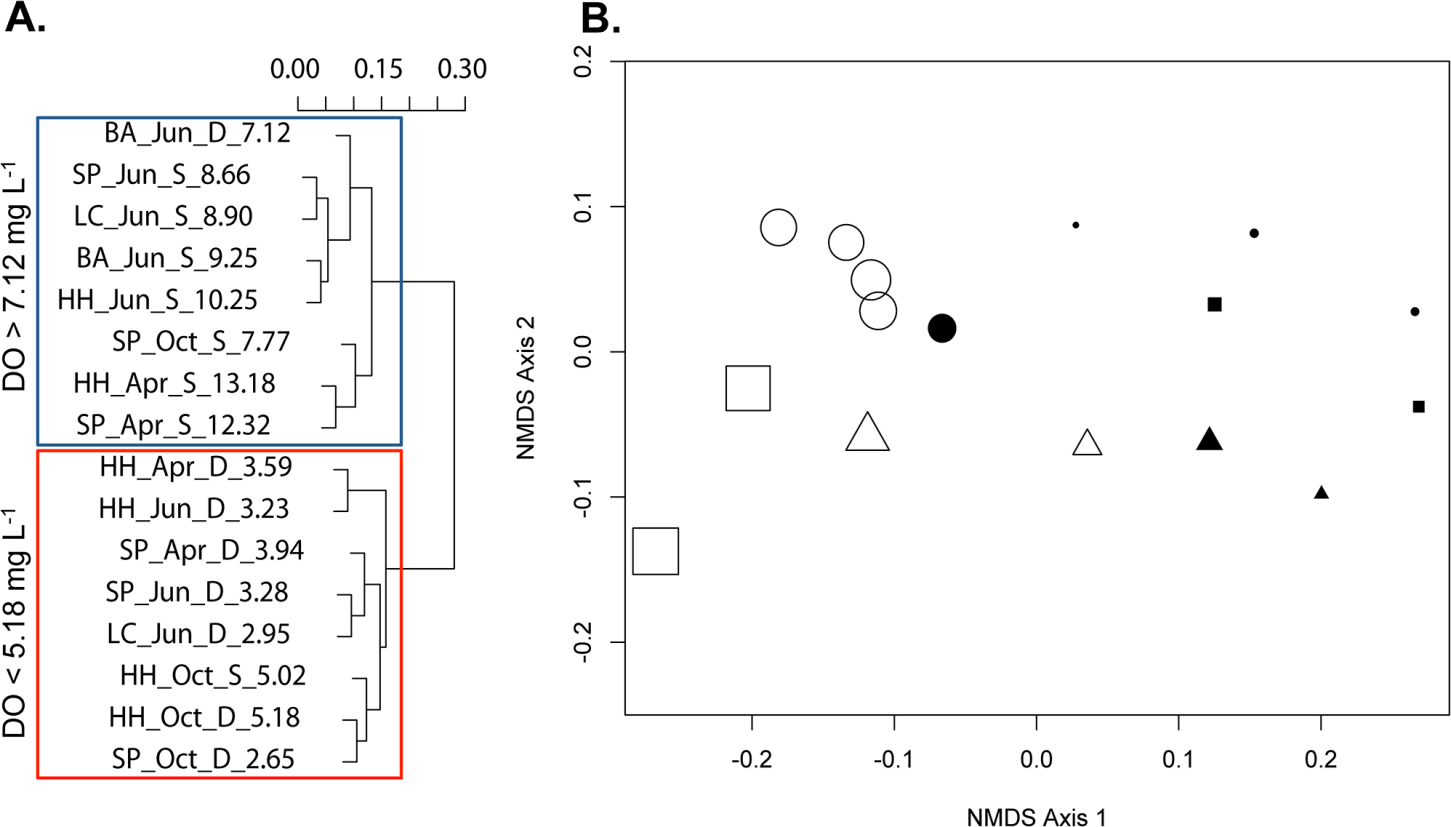
Both (A) parametric and (B) non-parametric analyses suggest that the bacterial community composition shifts between 5.18 and 7.12 mg O_2_ L^-1^. (A) Dendrogram of parametric hierarchical clustering approach using Sorensen-abundance based community composition dissimilarity. Numbers following Sample ID show the DO concentration in mg L^-1^. (B) Non-metric multidimensional scaling ordination shows bacterial community composition of samples are more similar to those from the same water depth, season, or DO concentration. Position of samples is calculated using the Sorensen abundance-based similarity index of bacterial community composition and the absolute dissimilarities between samples is condensed onto two-dimensions with a resulting stress value of 0.0679. Points are coded by water depth as either deep (filled symbols) or surface (open symbols) and by season as April (square), June (circle), or October (triangle). The size of the sample point is proportional to DO content with the highest DO represented by the largest sample point.

Change in bacterial community composition (BCC) was examined across all possible combinations of abiotic variables measured within this study, and DO concentration alone was most correlated with dissimilarities in the composition of bacterial communities among samples (Spearman’s rho = 0.7320, Table 2). Further, we examined the relevance of a threshold of DO concentration for bacterial communities in Hood Canal by applying three separate statistical tests: 1) a parametric assessment of the community data using hierarchical clustering, 2) a non-parametric assessment of the community data using an ANOSIM, and 3) an analysis of community thresholds determined by patterns of individual taxa using TITAN. Using a parametric clustering approach the BCC samples were plotted on a dendrogram based on the dissimilarity in BCC between pairs of samples. The clustering dendrogram reveals two distinct clusters of samples based solely on community composition data without any *a priori* knowledge of environmental factors (Fig 5A). Most of the surface water samples fell in the high DO cluster, except for the surface sample collected in October, which clustered with the lower DO samples as expected given the DO level at that sample was below 5.18 mg O_2_ L^-1^, the level that defined the low DO cluster. Similarly, all deep samples clustered with each other except for the deep sample collected in June from Bangor when DO concentrations were higher than 7.12 mg L^-1^. The two clusters suggest that DO is a strong factor in shaping BCC.

In the second analysis, the samples were again plotted based on the pairwise dissimilarities in BCC using non-parametric multidimensional scaling (NDMS). The NMDS further supports that two groups of samples exist and that these two groups are delineated by DO, rather than depth alone (Fig 5B). An analysis of similarity (ANOSIM) confirmed that depth and DO are significant descriptors of the two groups of samples, but the association of DO with community composition is stronger than the association with depth (ANOSIM: R_DO_ = 0.827, p_DO_ < 0.01; R_depth_ = 0.6144, p_depth_ < 0.01).

**Fig 6:**
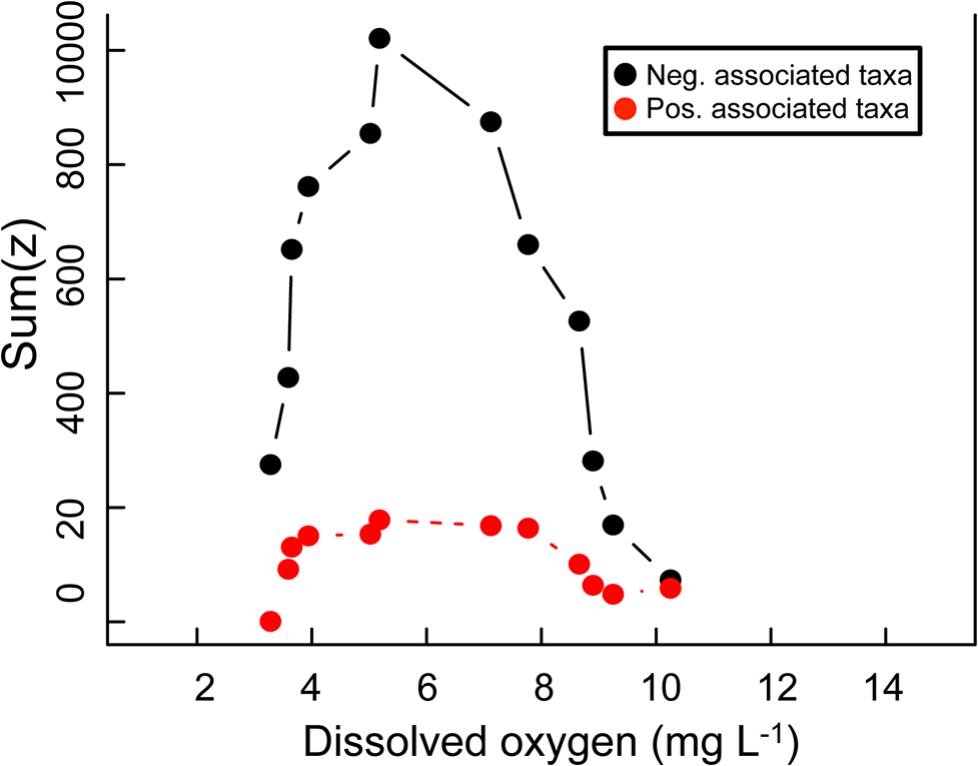
Bacterial community threshold analysis for DO in Hood Canal. Taxa-Z plot from TITAN showing the cumulative change points of taxa abundances across the DO gradient. The peak in change-point is considered an approximate community threshold for DO concentration. Z- are taxa that have a negative association with DO and Z+ are taxa that have a positive association with DO.

Lastly, we examined taxon-specific responses to the DO gradient to identify a threshold concentration of DO at which both positively and negatively-associated taxa change, thus giving a community-wide change point. TITAN identified a threshold value of 6.15 mg O_2_ L^-1^ for both positively and negatively associated taxa (Fig 6). This implies that a shift in BCC occurs at 6.15 mg O_2_ L^-1^ (95% CI [3.62, 8.78]) from a community dominated by taxa that are positively-associated with DO to a community dominated by negatively-associated taxa. Though the confidence interval surrounding the community change-point predicted by TITAN is broad, the other two independent statistical tests (hierarchical clustering and NMDS) also suggest a community change point existing between 5.18 mg O_2_ L^-1^ and 7.12 mg O_2_ L^-1^.

### Dominant taxa in Hood Canal, WA and indicator taxa of low DO

**Fig 7:**
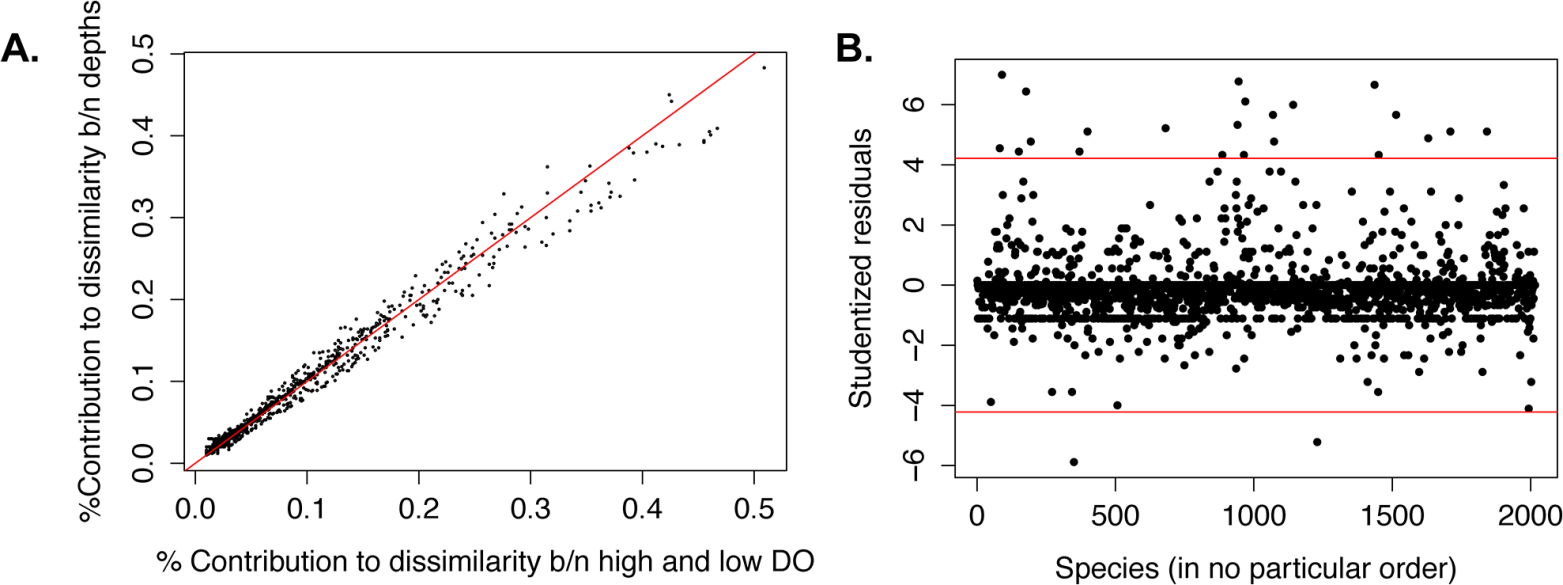
DO rather than depth affects taxa distributions and twenty-five taxa were identified as indicators of different DO conditions. (A) Plot of each OTU’s contribution to the dissimilarity between high and low DO groupings of samples versus surface and deep grouping of samples. Red line shows one-to-one line representing equal contribution to both DO effect and depth effect. Taxa falling to the right of the red one-to-one line contribute more to the dissimilarity between high and low DO groupings than depth groupings suggesting a stronger effect of DO than depth. (B) Plot showing the studentized residuals calculated for each OTU to identify significant deviation from the null hypothesis that there is no difference between the OTU’s contribution to dissimilarity between DO and depth groupings. Each red horizontal line shows the cutoff for significance. Points above the upper red line represent OTUs that contribute significant dissimilarity between DO groupings thus are indicators of high or low DO, whereas points below the lower red line are significant indicators of a depth effect.

The relative proportions of the fifteen most abundant bacterial classes changed significantly as DO dropped below 6 mg O_2_ L^-1^ (Fig 4, G-test; G = 44.564, p < 0.01). Shifts in the most abundant bacterial classes prompted further investigation into the individual taxa that may be representative of changing DO conditions. By comparing the abundances of taxa across our samples using pyrosequencing data, we identified taxa that may be indicators of low DO in Hood Canal. We identified 23 taxa that had significantly higher DO SIMPER values than depth values at an alpha level of 0.05 (Fig 7A and 7B), suggesting that these taxa drive dissimilarity between high and low DO communities and can thus be considered indicators of different DO conditions. Only two taxa identified from SIMPER analysis were indicators of high DO waters in Hood Canal. One of these taxa, Alphaproteobacteria_03_61, was most similar to members of the family Rhodobacteriacaea that are commonly detected in marine surface waters, particularly during phytoplankton blooms (S2 Table). The remaining 21 taxa were indicators of low DO waters in Hood Canal. The majority of the low DO indicator taxa were common marine bacterial groups including Rhodobacteracaea, SAR11, SAR406, *Nitrospina*, *Methylobacter*, and SAR86, which are often found across a wide range of marine habitats (S2 Table). These indicator taxa were abundant OTUs throughout the entire dataset but have significant shifts with decreasing DO, suggesting that these OTUs may be specialized members of common, ubiquitous groups that have undergone niche differentiation.

## Discussion

On a global scale, DO content in coastal waters has decreased in recent decades and is arguably becoming one of the most important indicators of aquatic ecosystem health [1]. We investigated changes in bacterial community diversity and composition along a DO gradient in Hood Canal, WA, and, for multiple indices of bacterial community structure, we found a role for DO that was stronger than that of the other abiotic factors assessed here. Further, we identified a threshold concentration of DO at which the bacterial community composition shifted significantly, suggesting that the structure of bacterial communities is intimately linked to DO.

A strong negative relationship between bacterial richness and DO was detected across all samples (Fig 3), which highlights a pattern consistent with other systems including the seasonally anoxic Canadian fjord, Saanich Inlet [21] and in the open ocean water column in the Eastern Tropical North Pacific [12]. However, when DO concentration falls below hypoxic levels in Saanich Inlet and the Eastern Tropical North Pacific, bacterial richness has been shown to decrease sharply. Expanding our sampling gradient to extremely low DO conditions periodically present in Hood Canal could elucidate such a potential unimodal relationship between DO and richness (Fig 3). Indirect effects of oxygen-depletion on the bacterial community at low DO could explain the increasing richness at low DO. For instance, energy is transferred from macrofauna to microbes as oxygen is depleted as oxygen-dependent organisms either avoid hypoxic areas or die [1,8,49]. This transfer of energy to the bacterial community as DO decreases could also allow for higher taxa richness exemplifying a common theme in ecology where increasing bioavailable energy allows for coexistence of multiple taxa resulting in greater biodiversity [50–52].

Dissolved oxygen played a strong role in shaping bacterial community composition, as well. Our data suggest a significant shift in bacterial community composition at a threshold DO concentration between 5.18 and 7.12 mg O_2_ L^-1^, which is not only higher than the published thresholds of 2–4 mg O_2_ L^-1^ for higher trophic-level organisms [2], but also much higher than the minimum concentration for aerobic metabolisms (< 96 ng O_2_ L^-1^) [53,54] as well as the maximum DO concentration for denitrification (160 μg O_2_ L^-1^) [55]. Therefore, the direct effects of oxygen on bacteria may not be the cause of the compositional shift we detected. Alternatively, indirect effects through trophic interactions including altered bacterial grazing or viral infection may favor a different bacterial community, as has been shown under other climate change scenarios such as increased temperature and UV radiation [56]. Additionally, abiotic factors not measured in this study that may co-vary with DO may be causing a compositional shift. For example, the composition and quality of dissolved organic matter can strongly influence bacterial community composition [57]. Nonetheless, evidence for significant changes to the bacterial community at threshold DO concentrations above the conventional definition of hypoxia suggests that impacts of decreasing DO have implications for ecosystem processes and health before sublethal and lethal effects on macrofauna are detected.

We identified 21 indicator taxa of low DO that were present across the majority of samples spanning a full range of DO, but their relative abundances change significantly at the threshold DO concentration. These low-DO indicator taxa were identified as members of common and ubiquitous marine bacteria including *Roseobacter*, SAR11, SAR202, SAR406, SAR86, OM190, and *Nitrospina*, which are often detected in coastal regions, estuaries, upwelling zones, and other pelagic oxygen minimum zones (S2 Table). Other OTUs from these common marine groups were also detected throughout our dataset, but were not identified as indicators of low DO. This may suggest that particular low-DO adapted ecotypes of common marine bacteria exist, highlighting the taxonomic and functional redundancy of bacterial communities [58]. Ecotypes of SAR11 are known to be distributed across chemical and physical gradients in marine surface waters suggesting that niche separation has led to the diversification within this group [59,60]. It is possible that similar mechanisms are responsible for shaping the distribution of indicator taxa we detected. Further research into the indicator taxa we detected here will elucidate the adaptations of these potential ecotypes that are responsible for providing a competitive advantage at low DO.

Our initial survey across a gradient of spatial and temporal conditions in Hood Canal has provided a snapshot into the complex relationship between bacterial community structure and DO. An obvious limitation of this study is that a few discrete samples do not capture fine-scale patterns or more extreme hypoxia conditions; yet with even limited sampling we were able to detect a significant shift in community composition at a threshold level of DO. Expanded sampling in both space and time will aid in refining this threshold concentration and the mechanisms for compositional shifts. The long residence time of water in Hood Canal [25] combined with the reduced grazing pressure on bacteria at depth or lower DO as a result of a shift in the distribution of higher trophic levels where DO is depleted [31] could also allow for the detection of more bacterial taxa, many of which may be in a dormant or inactive state. Dormancy can contribute to high taxonomic diversity and also act as a reservoir of functional diversity that can be resuscitated under favorable conditions [61].

As bacterial communities shift in diversity and composition, it is likely that the bacterially mediated nutrient and energy cycles also change, which has implications for changes in global cycles as oxygen minimum zones continue to expand. The hydrography of Hood Canal generates a wide gradient of abiotic factors including DO that change on an annual scale, which establishes a unique natural laboratory in which these questions can be explored. We are only beginning to understand how microbial communities are responding to decreasing DO, but further questions into the effects on individual functional groups of Bacteria will be crucial in predicting the global consequences of expanding oxygen minimum zones.

## Supporting Information

S1 Fig Rarefaction curves of (A) All 16S sequences, (B) Alphaproteobacterial sequences only, (C) Gammaproteobacterial sequences only, and (D) Flavobacterial sequences only. Each solid line represents the sequence accumulation curve for each sample in the dataset. The dotted line represents a 1:1 line, which is the maximum taxa accumulation per sequence. The dashed, vertical line in each plot represents the number of sequences to which each set was subsampled. Note that plot A is on a different scale from plots B-D.
(TIF)

S2 Fig Depth profiles of physical and chemical data recorded during sampling. (A) April sampling at Hama Hama and Sister’s Point, (B) during June sampling at all stations, and (C) during October sampling at Hama Hama and Sister’s Point.
(PDF)

S1 Table Abiotic data collected to accompany each bacterial pyrosequencing sample and used in PCA and regression analyses. (PDF)

S2 Table Indicator taxa of high or low DO conditions in Hood Canal, WA, as identified by SIMPER analysis, and their associated top BLAST matches on NCBI. “Contrib.depth” and “Contrib.DO” describe the relative contribution of a particular taxon to the dissimilarity between sample groups defined by water depth or DO content, respectively. “Hi” or “Lo DO ave abund” describes the average abundance of a particular taxon in the samples grouped as either high or low DO, respectively.
(XLSX)
