# Supplementary figures and images for: A Dissolved Oxygen Threshold for Shifts in Bacterial Community Structure in a Seasonally Hypoxic Estuary
Source: PLoS One. 2015 Aug 13;10(8):e0135731. doi: 10.1371/journal.pone.0135731 (PMC4535773; doi:10.1371/journal.pone.0135731)

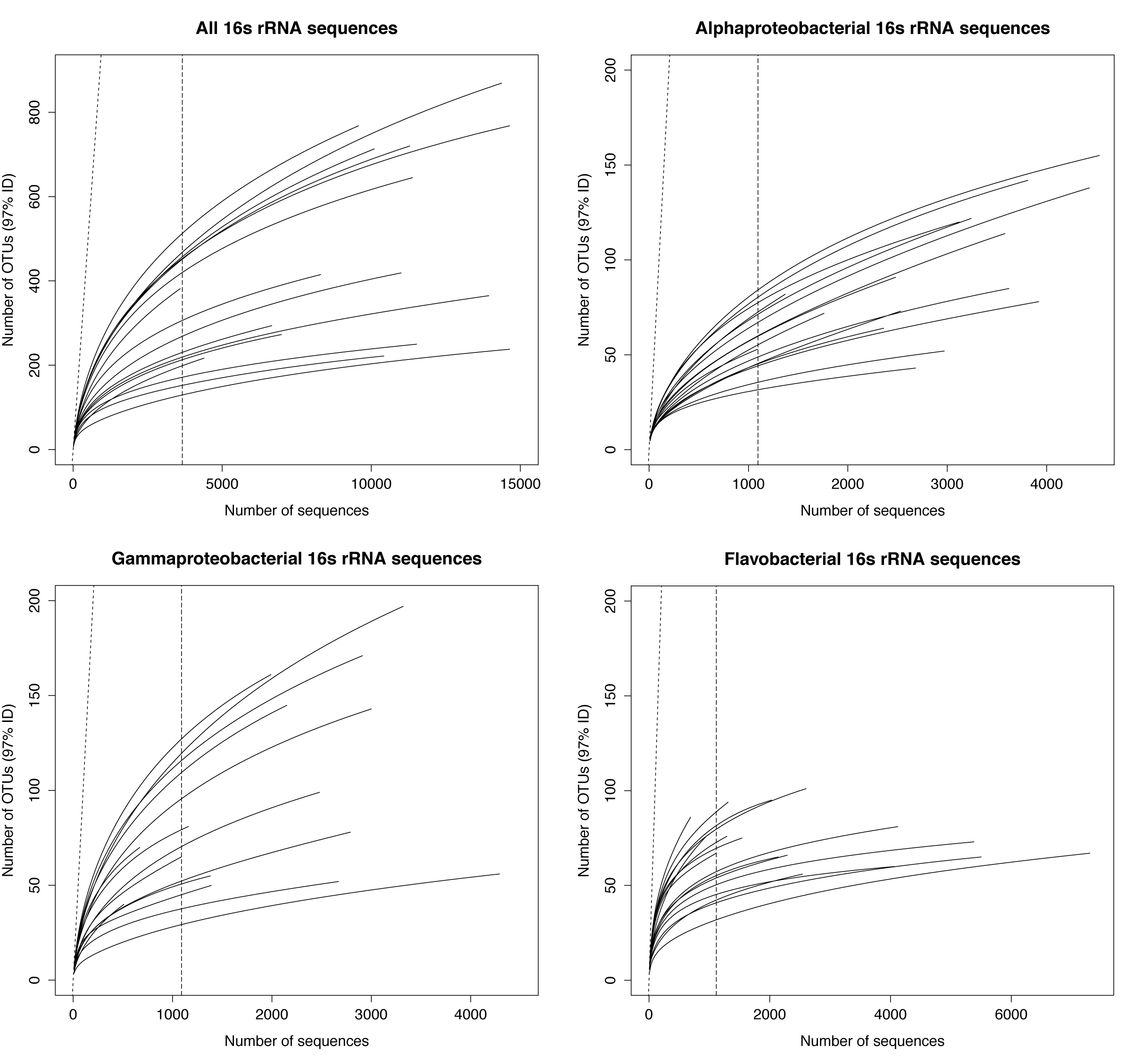

Supplement: S1 Fig — Each solid line represents the sequence accumulation curve for each sample in the dataset. The dotted line represents a 1:1 line, which is the maximum taxa accumulation per sequence. The dashed, vertical line in each plot represents the number of sequences to which each set was subsampled. Note that plot A is on a different scale from plots B-D. (TIF) [file pone.0135731.s001.tif]
