# Supplementary material for: A Dissolved Oxygen Threshold for Shifts in Bacterial Community Structure in a Seasonally Hypoxic Estuary
Source: PLoS One. 2015 Aug 13;10(8):e0135731. doi: 10.1371/journal.pone.0135731 (PMC4535773; doi:10.1371/journal.pone.0135731)

April 2007

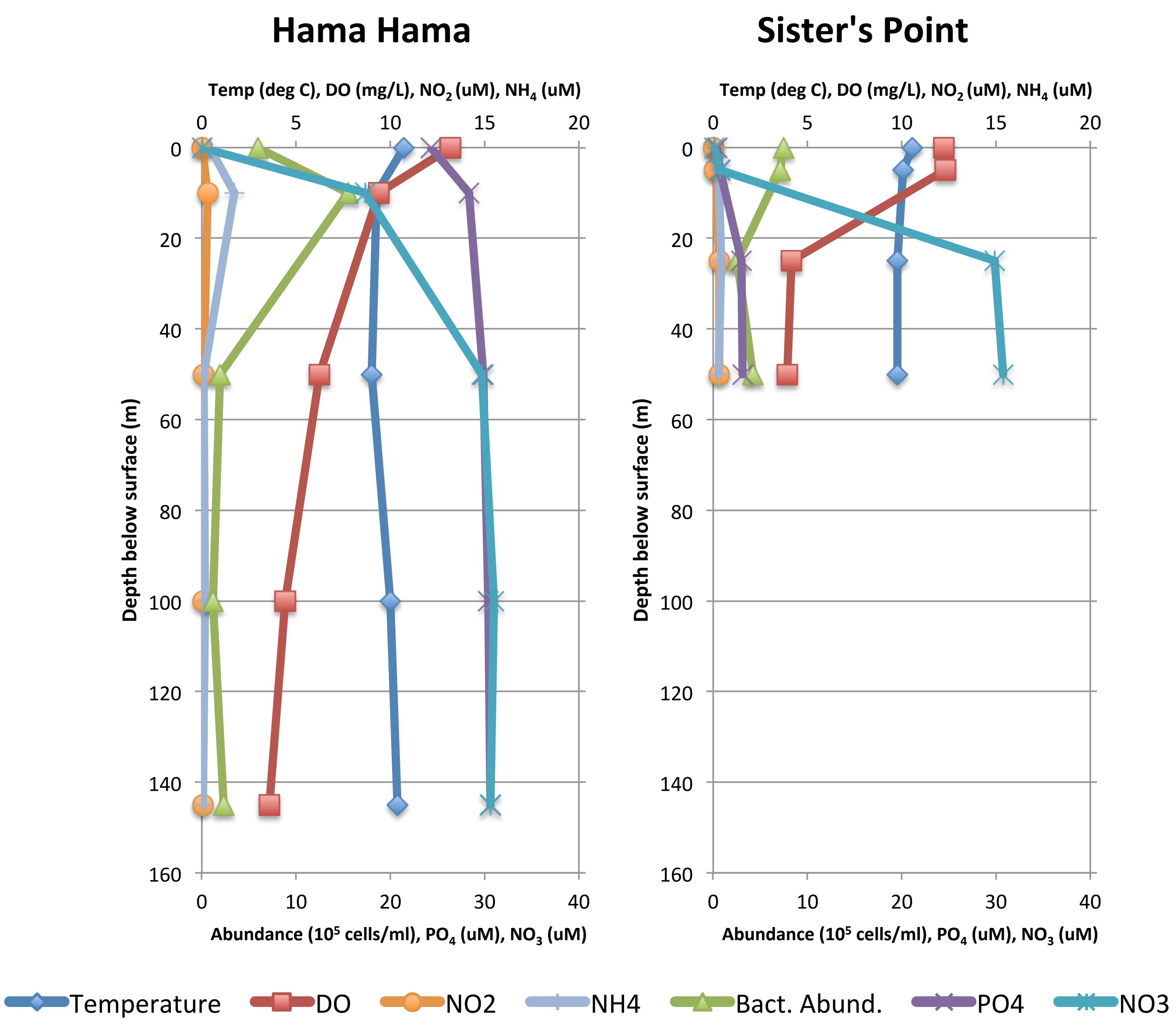

Hama Hama
Sister's Point
Temp (deg C), DO (mg/L), NO2 (uM), NH4 (uM)
0
5
10
15
20
Depth below surface (m)
0
20
40
60
80
100
120
140
160
0
10
20
30
40
Abundance (10^5 cells/ml), PO4 (uM), NO3 (uM)
Temperature
DO
NO2
NH4
Bact. Abund.
PO4
NO3

June 2007

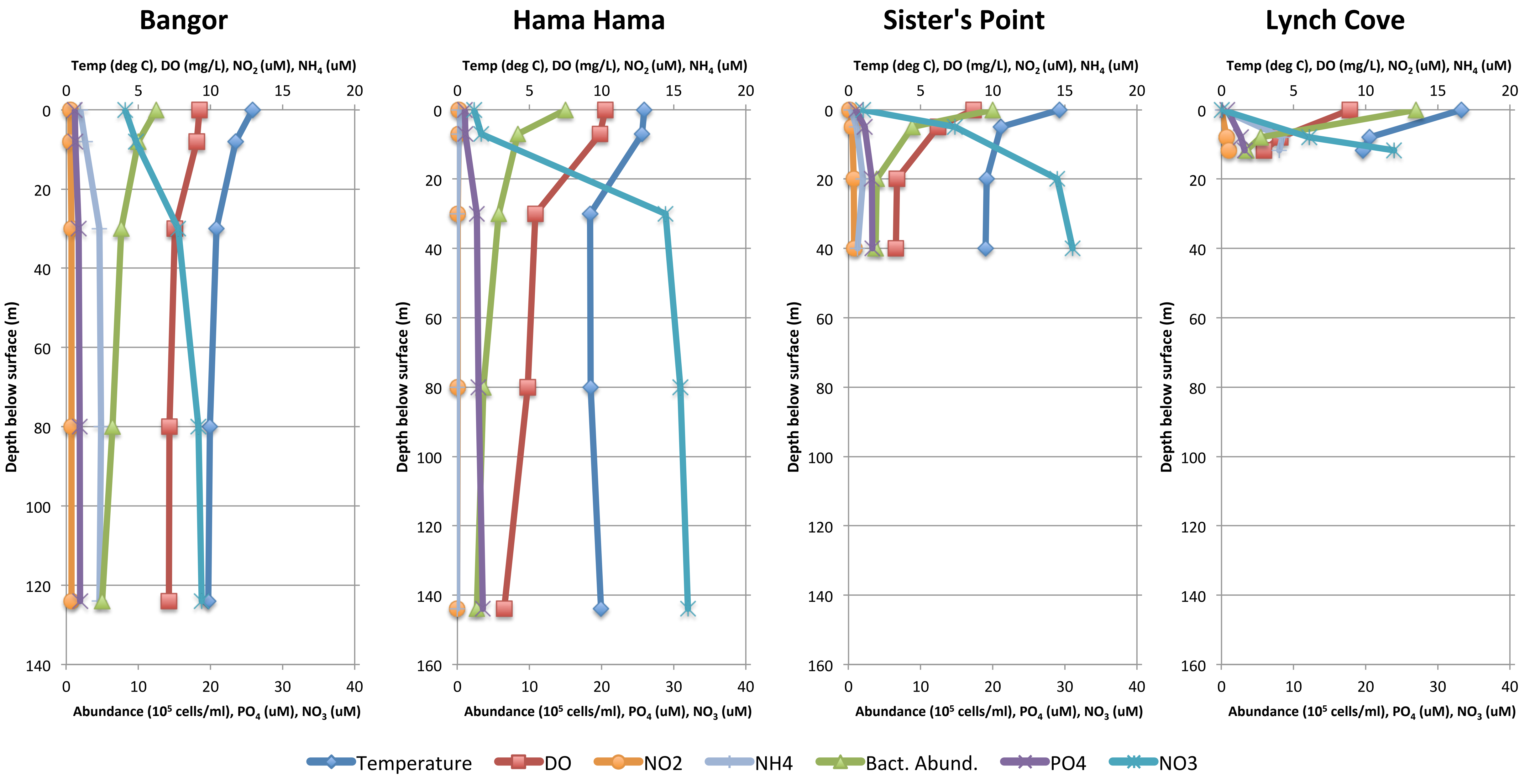

Bangor
Hama Hama
Sister's Point
Lynch Cove
Temp (deg C), DO (mg/L), NO_2 (uM), NH_4 (uM)
Depth below surface (m)
Abundance (10^5 cells/ml), PO_4 (uM), NO_3 (uM)
Temperature
DO
NO2
NH4
Bact. Abund.
PO4
NO3

October 2007

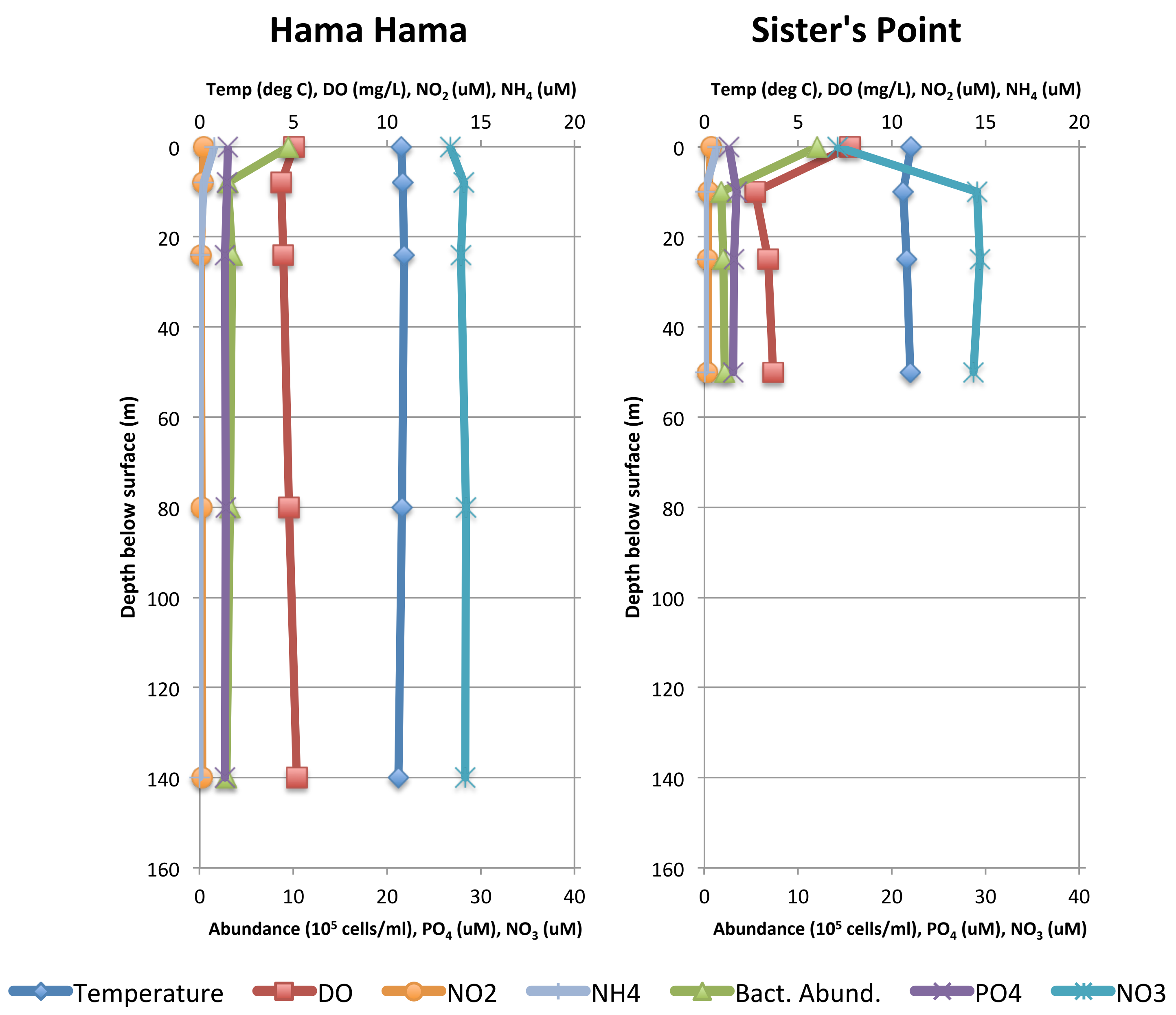

Supplement: S2 Fig — (A) April sampling at Hama Hama and Sister’s Point, (B) during June sampling at all stations, and (C) during October sampling at Hama Hama and Sister’s Point. (PDF) [file pone.0135731.s002.pdf]
