# Supplementary material for: A Dissolved Oxygen Threshold for Shifts in Bacterial Community Structure in a Seasonally Hypoxic Estuary
Source: PLoS One. 2015 Aug 13;10(8):e0135731. doi: 10.1371/journal.pone.0135731 (PMC4535773; doi:10.1371/journal.pone.0135731)
Supplement: S1 Table — (PDF) [file pone.0135731.s003.pdf]

Location	Depth (m)	Nitrate [μM] Apr	Nitrate June	Nitrate Oct	Nitrite [μM] Apr	Nitrite June	Nitrite Oct	Ammonium [μM] Apr	Ammonium June	Ammonium Oct	Phosphate [μM] Apr	Phosphate June	Phosphate Oct	Silicate [μM] Apr	Silicate June	Silicate Oct	Salinity [PSU] Apr	Salinity June	Salinity Oct	Temperature [°C] Apr	Temperature June	Temperature Oct	Oxygen [mg L^{-1}] Apr	Oxygen June	Oxygen Oct
	0		8.18			0.30			0.96			1.18			27.54			28.56			12.91			9.25	
**Bangor**	124		18.79			0.38			2.31			1.91			41.85			30.53			9.86			7.12	
	0	0.73	2.32	26.77	0.00	0.08	0.21	0.46	0.10	0.77	0.01	1.01	3.03	36.52	27.41	79.97	24.29	27.72	28.93	10.71	12.96	10.74	13.18	10.25	5.02
**Hama- Hama**	140	30.76	31.97	28.36	0.05	0.00	0.14	0.06	0.00	0.00	3.46	3.55	2.71	77.90	80.30	58.60	30.62	30.32	30.66	10.39	9.95	10.62	3.59	3.23	5.18
	0	0.03	2.00	14.24	0.04	0.05	0.37	0.30	0.16	0.67	0.73	1.00	2.62	56.23	46.75	91.08	23.59	26.18	26.49	10.09	14.63	11.03	12.32	8.66	7.77
**Sisters Point**	45	30.59	31.08	28.69	0.32	0.38	0.17	0.28	0.62	0.01	3.12	3.34	3.10	73.79	75.04	65.39	29.84	29.71	30.49	9.78	9.51	10.97	3.94	3.28	3.65
	0		0.08			0.00			0.04			0.77			41.47			24.80			16.62			8.90	
**Lynch Cove**	11.7		23.94			0.50			4.01			3.21			73.64			29.53			9.80			2.95	
